# Aggressiveness of non-EMT breast cancer cells relies on FBXO11 activity

**DOI:** 10.1186/s12943-018-0918-6

**Published:** 2018-12-10

**Authors:** Sofie Otzen Bagger, Branden Michael Hopkinson, Deo Prakash Pandey, Mads Bak, Andreas Vincent Brydholm, Rene Villadsen, Kristian Helin, Lone Rønnov-Jessen, Ole William Petersen, Jiyoung Kim

**Affiliations:** 1 Department of Cellular and Molecular Medicine, Faculty of Health Sciences, University of Copenhagen, Blegdamsvej 3, DK-2200 Copenhagen N, Denmark; 2 Novo Nordisk Foundation Center for Stem Cell Biology (DanStem), University of Copenhagen, Blegdamsvej 3, DK-2200 Copenhagen N, Denmark; 3 Biotech Research and Innovation Centre (BRIC), University of Copenhagen, Ole Maaløes Vej 5, DK-2200 Copenhagen N, Denmark; 4 Section for Cell Biology and Physiology, Department of Biology, Faculty of Science, University of Copenhagen, Universitetsparken 13, DK-2100 Copenhagen Ø, Denmark; 5 Department of Molecular Microbiology, Oslo University Hospital, Sognsvannsveien 20, NO-0372 Oslo, Norway

**Keywords:** Breast cancer, shRNA screening, Collective migration, Non-EMT

## Abstract

Tumorigenesis is increasingly considered to rely on subclones of cells poised to undergo an epithelial to mesenchymal transition (EMT) program. We and others have provided evidence, however, that the tumorigenesis of human breast cancer is not always restricted to typical EMT cells but is also somewhat paradoxically conveyed by subclones of apparently differentiated, non-EMT cells. Here we characterize such non-EMT-like and EMT-like subclones. Through a loss-of-function screen we found that a member of the E3 ubiquitin ligase complexes, FBXO11, specifically fuels tumor formation of a non-EMT-like clone by restraining the p53/p21 pathway. Interestingly, in the related EMT-like clone, FBXO11 operates through the BCL2 pathway with little or no impact on tumorigenesis. These data command caution in attempts to assess tumorigenesis prospectively based on EMT profiling, and they emphasize the importance of next generation subtyping of tumors, that is at the level of clonal composition.

**Electronic supplementary material:**

The online version of this article (10.1186/s12943-018-0918-6) contains supplementary material, which is available to authorized users.

In recent years, due to its aggressive behavior, much attention has been directed towards the basal-like subtype of breast cancer believed to be caused by cancer stem cell activity often considered synonymous to the concept of EMT. Accordingly, as a consequence of its relatively differentiated, non-EMT-like appearance, the most frequent subtype of breast cancer, the luminal, has been somewhat understudied in this regard. Indeed, the very impact of EMT on breast cancer has been questioned altogether [1, 2]. With the purpose of examining cancer stem cell activity among typical epithelial cells in breast cancer we have previously focused on comparisons between clonally related non-EMT-like and EMT-like cells [3]. To our surprise we found that independently of subtype the non-EMT-like cells are more tumorigenic and tumor-initiating than the EMT-like cells [3, 4]. When we cloned cells with a stem-like profile, we found that they readily generated luminal-like progeny to indicate the existence of a hierarchy which could sustain heterogeneity as previously described for human breast cancer [3]. We also cloned differentiated variants without a typical stem-cell profile, but with a luminal profile which resembles the majority of grade I luminal breast cancers, i.e. polarized luminal-like cells without signs of basal traits. While the latter clones turned out to be both highly tumor-initiating and invasive [3], the question remained open as to how aggressiveness is maintained in such clones if not by hijacked stem-cell or established EMT-related pathways.

Large-scale loss-of-function screens have been successfully applied to identify tumorigenic mechanisms and in turn have led to discovery of novel targets for drug intervention [5]. Here, we performed an shRNA screen in non-EMT-like and EMT-like clones for identifying differentially depleted shRNAs and found significantly depleted shRNAs in a clone-dependent manner. Based on this, we propose to reappraise pathways of tumorigenesis in non-EMT breast cancer subclones and to emphasize clonal heterogeneity as a supplement to breast cancer subtyping.

## Characterization of MCF7 breast cancer cell clones as a model of tumor aggressiveness and FBXO11 as a functional readout

**Fig. 1:**
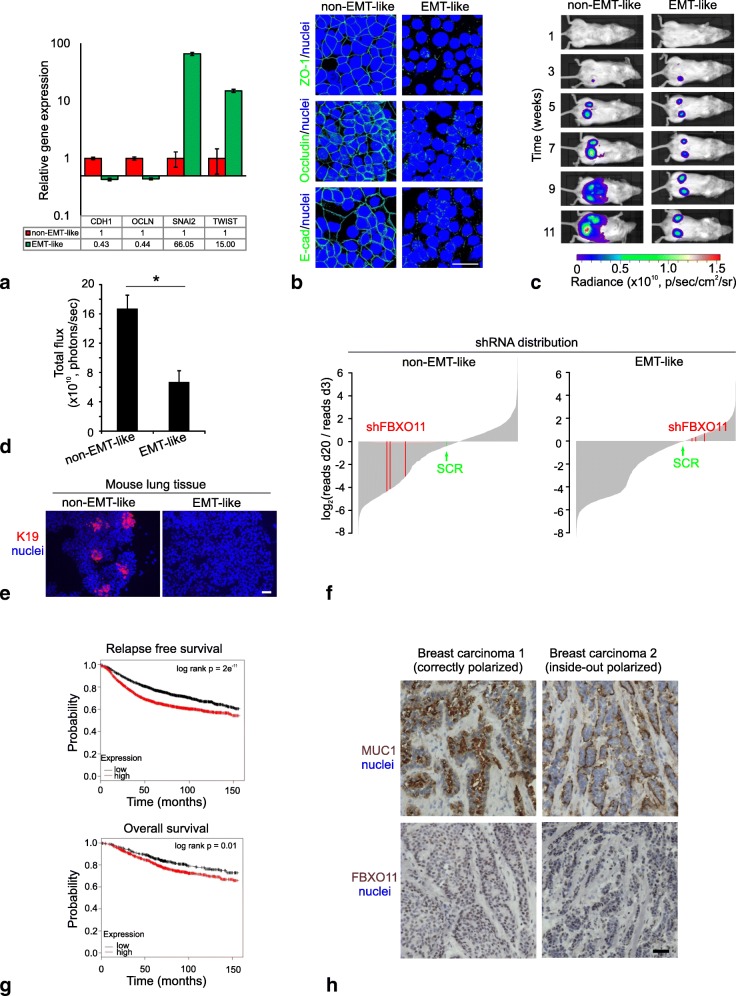
Characterization of MCF7 breast cancer cell clones as a model of tumor aggressiveness and FBXO11 as a functional readout. **a** RT-qPCR analysis of relative EMT marker expression shows upregulation of epithelial markers CDH1 and OCLN in non-EMT cells and upregulation of mesenchymal markers SNAI2 and TWIST in EMT-like cells. E-cadherin is encoded by CDH1; occludin by OCLN. Error bars represent SD of quadruplicates. **b** Monolayer culture non-EMT-like (left column) and EMT-like (right column) MCF7 clones stained (green) with ZO-1 (upper row), Occludin (middle row), E-cadherin (lower row), and DAPI (blue, nuclei). Only the non-EMT-like clone exhibits distinct staining at the cell-cell junctions. Bar = 50 μm. **c** Representative BLI of NOG mice inoculated with 10^4^ non-EMT-like (left column) or EMT-like (right column) MCF7-pFU-L2G cells (*n* = 6 inoculations/cell-type). Time indicates weeks after inoculation. Non-EMT-like cells grow to a larger size than the EMT-like cells. **d** Quantification of tumor sizes measured as total flux by in vivo BLI at week 11 (n = 6 injections/group) (asterisk indicates *p* < 0.005 tested by t-test). Error bars represent SEM. **e** Representative immunofluorescence staining of human-specific K19 (red) on mouse lung sections reveals non-EMT-like-derived metastases (*n* = 4 mice/group). Metastatic colonies stained with K19 are only found in lungs of mice injected with non-EMT-like cells. Nuclei (blue) and scale bar, 50 μm. **f** Relative shRNA distributions in Non-EMT-like (left) and EMT-like (right) MCF7 cells 20 days after transduction with a lentiviral human-specific epigenetic shRNA library (referred as epi-library) in ascending order. The result is representative of 2 replicates. Negative controls, SCRs, are indicated by green and shFBXO11s in red. shFBXO11s are the most depleted shRNAs in the non-EMT-like cells compared to the EMT-like cells. **g** Kaplan-Meier plots of RFS (top) and OS (bottom) of breast cancer patients stratified by mRNA expression of FBXO11 using a web based survival analysis tool (kmplot.com). A total of 30 cohorts (3951 patients) are split into low (*n* = 1861) vs. high (*n* = 2090) expression groups for RFS (Hazard ratio (HR) = 1.46 (1.31–1.63), log rank *p* = 2 × 10^− 11^), and 11 cohorts (1402 patients) are split into low (*n* = 468) vs. high (*n* = 934) expression groups for OS (HR = 1.37 (1.08–1.74), log rank *p* = 0.01). **h** FBXO11 stains strongly in highly differentiated breast carcinomas. Representative immunostaining (brown) of MUC1 (top) or FBXO11 (bottom) out of six highly differentiated luminal breast carcinomas. Biopsy 1 is correctly (left panel) and biopsy 2 is inversely polarized (right panel) based on MUC1 expression and both express FBXO11 in the nuclei. Nuclei (blue) and scale bar, 50 μm

In order to investigate mechanisms alternative to EMT, which facilitate tumor progression, we used an established set of non-EMT-like and EMT-like clones of MCF7 cells known to differentially express CDH1 and TWIST, SNAI2, FN and VIM, respectively [3]. Here, using an RT-qPCR approach different from the one used previously, the EMT properties of these cells are further substantiated (Fig. 1a). Also, we confirm the epithelial properties of the non-EMT-like clone by positive staining for ZO-1, Occludin and E-cadherin, whereas the EMT-like clone expresses little of these markers (Fig. 1b). Notably, transplantation to NOG mice for in vivo imaging resulted in a significant increase in tumor size of the non-EMT-like clone compared to the EMT-like (Fig. 1c and d). Immunofluorescence imaging with keratin 19 further revealed that only non-EMT-like cells formed metastatic colonies in the lungs (Fig. 1e). Since MCF7 breast cancer cells are considered quite stationary in monolayer culture [6], we did not pursue migration at the single-cell level as a readout of EMT. These findings nevertheless underscore that cancer-cell aggressiveness is not restricted to cells with a typical EMT-like profile, emphasizing the need for alternative explanations to this behavior.

Accordingly, we applied a loss-of-function screen with a lentiviral library of 2365 pLKO shRNA constructs (Fig. 1f and Additional files 1 and 2). While the screen identified several potentially interesting genes significantly depleted in both clones, here we focused on differently depleted shRNAs. Among these were FBXO11, NAT8L and TOX4 in the non-EMT-like and NAP1L4, TCF20 and ID3 in the EMT-like clones. Since the non-EMT-like clones are more tumorigenic and metastatic, we selected FBXO11 for further scrutiny (Fig. 1f).

FBXO11 has been reported to have different cellular roles. On one hand, it has been shown to keep EMT in check by facilitating degradation of SNAI1 and SNAI2 (reviewed in [7]). On the other hand, FBXO11 has also been reported to neddylate p53 protein, leading to inhibition of its activity [8]. Since p53 is a bona fide tumor suppressor, a consequence of FBXO11 expression in this context would be to promote tumor growth. Accordingly, we show that based on multiple cohorts [9], FBXO11 is a robust predictor of a poor clinical outcome in breast cancer (Fig. 1g). To validate the relevance of FBXO11 specifically in luminal breast cancer which is the topic of the present study, we examined 30 ER-positive breast cancer biopsies for FBXO11 expression. We found nuclear staining with FBXO11 in the majority of the cancer cells in 18 biopsies and six were highly differentiated, polarized, tubular breast carcinomas (Fig. 1h), providing proof of principle that tumors with FBXO11 expression are not necessarily linked to EMT. These data were supported by multivariate analysis of FBXO11 expression and impact on survival, specifically within the subtype of luminal breast cancer (Additional file 3: Table S1). Based on these findings, FBXO11 signaling was further investigated with respect to tumorigenicity in non-EMT breast cancer cells.

## shFBXO11 preferentially attenuates tumor initiation of non-EMT breast cancer cells

**Fig. 2:**
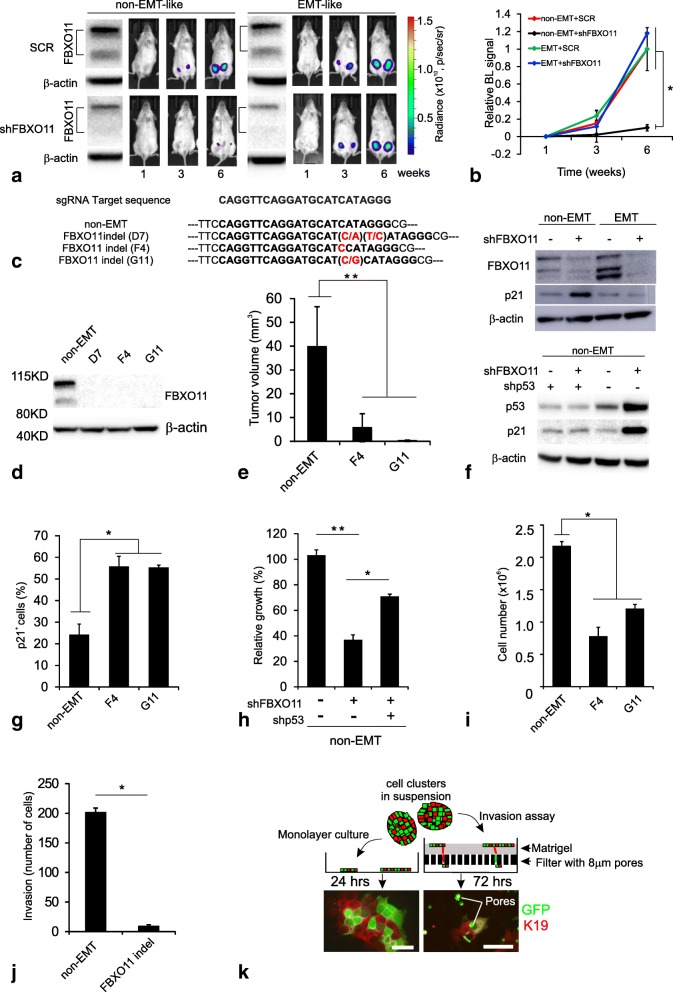
shFBXO11 preferentially attenuates tumor initiation of non-EMT-like cells. **a** Non-EMT-like cells (left) and EMT-like cells (right) transduced with SCR or shFBXO11 and inoculated in NOG mice (10^3^ cells per injection, *n* = 6 to 10 injections/group). Western blots (first columns of each panel) illustrate FBXO11 and β-actin expression in inoculated cells. Representative bioluminescent (BL) signal of mice shows a delay in tumor initiation only in mice inoculated with shFBXO11 non-EMT-like cells. **b** Relative quantification of tumor growth illustrated in (**a**). BL signals of each clone are measured as total flux and normalized by total flux of its corresponding SCR-transduced clone at week 6. Tumor growth is significantly inhibited only in the FBXO11-depleted non-EMT-like cells (asterisk indicates *p* < 0.05 tested by ANOVA with Tukey’s test). Error bars represent SEM. **c** Sanger sequencing confirming three different FBXO11 indel clones (D7, F4 and G11) as compared to sgRNA target sequence of the CRISPR-Cas9 gene-editing system. Intact sequence is shown in non-EMT cells and inserted nucleotides are marked in red. F4 clone gained the same indel mutations in both alleles. **d** Western blot shows that FBXO11 indel clones do not express FBXO11 proteins. **e** Tumor volume of non-EMT and FBXO11 indel clone xenografts as measured in week 6 after injection of 10^3^ cells (n = 6 injections/group, asterisks indicate *p* < 0.00005 by ANOVA with Tukey’s test). Error bars represent SD. **f** Western blot of whole lysates isolated from non-EMT-like- and EMT-like cells transduced with SCR or shFBXO11 and stained for FBXO11 and p21 shows that p21 is exclusively induced by shFBOX11 in non-EMT-like cells (upper). Western blot of whole lysates of non-EMT-like cells transduced with either or both of shFBXO11 and shp53 and stained for p53, p21, and β-actin. shFBXO11-mediated induction of p21 relies on p53 protein (lower). **g** FBXO11 indel clones significantly induce p21 protein. The percentage of immunostained p21^+^ cells in a total of approximately 1000 cells, was automatically counted with image J in triplicates (asterisks indicate *p* < 0.0005 by ANOVA with Tukey’s test). Error bars represent SD. **h** Quantification of cell proliferation of GFP^+^ cells as influenced by shFBXO11 and shp53 shows that the shFBXO11-induced growth reduction can be partly rescued by shp53 (asterisks ** and * indicate *p* < 0.0001 and *p* < 0.005 by t-test, respectively). Error bars represent SD. **i** Quantification of cell number upon FBXO11 knockout in non-EMT-like cells shows that cell number in culture is significantly reduced by FBXO11 knockout. Equal numbers (10^5^) of two different FBXO11 indel clones (G11 and F4) or the control cells (non-EMT-like) were cultured for 8 days prior to cell-counting (asterisk indicates *p* < 0.01 tested by ANOVA with Tukey’s test). Error bars, SD. **j** Quantification of invasive capacity of 5 × 10^4^ control non-EMT-like cells or a FBXO11 indel clone, F4 on Matrigel-coated filters in 72 h. Note that invasion is significantly abolished by deletion of FBXO11 (asterisk indicates *p* < 0.05 by t-test). Error bars represent SD of averages of quadruplicates in two independent experiments. **k** Depiction of the model used for testing in vitro collective migration (top) and cell invasion (bottom right). The clusters of mixture of GFP-labeled and unlabeled non-EMT-like cells (1:1 ratio) were either plated in an adhesion culture for 24 h (hrs) (bottom left) or plated in an invasion assay incubated for 72 h (bottom right), followed by staining for K19 (red) and GFP (green). Dual fluorescence imaging allows for the demonstration of doublets of green/red cells migrating through individual pores by collective migration. Scale bars, 50 μm

We first tested the knockdown efficiency of shRNAs targeting FBXO11 (shFBXO11s). As expected, in both clones FBXO11 was reduced by shFBXO11 (Additional file 3: Figure S1A from which selected lanes were included in Fig. 2a, and S1B). Next, we tested the consequences of silencing FBXO11 in terms of tumorigenicity in vivo. While tumor formation by the non-EMT-like cells was significantly inhibited by shFBXO11, no effect was recorded by the EMT-like cells (Fig. 2a and b). Similar data were obtained with a pGIPZ shFBXO11 (data not shown). To corroborate the effects of shFBXO11 in the non-EMT-like cells, we generated FBXO11 indel clones, using a CRISPR-Cas9 system (Fig. 2c and d), and further validated that FBXO11 is necessary for tumorigenesis (Fig. 2e). In summary, FBXO11 impacts primarily on tumorigenicity of non-EMT cells.

Given the known effect of FBXO11 on p53 and the role of a p53/p21/BCL2 pathway on invasion [7], we next investigated whether the FBXO11-dependent inhibition of non-EMT-like cells could be explained within such context. While the clinical datasets were too small to resolve a potential impact of FBXO11 on e.g. p53 (Additional file 3: Table S1), in culture, shFBXO11 led to a strong increase in p21 expression in the non-EMT-like cells but not in the EMT-like cells (Fig. 2f upper, and Additional file 3: Figure S1C and D). Similar data of p21 induction were obtained with the non-EMT-like cells deleted for FBXO11 (Fig. 2g). Importantly, the shFBXO11-induced p21 expression was abolished by co-transduction with shp53 (Fig. 2f lower). Also functionally, shFBXO11 significantly reduced the cell number to 37%, which was rescued in part, up to 71%, by concurrent silencing of p53 (Fig. 2h). These data were substantiated with two different FBXO11 indel clones (Fig. 2i). In contrast to the non-EMT cells, the p53/p21 pathway was not appreciably affected by FBXO11 in the EMT cells. Rather in these cells, FBXO11 led to an increase in BCL2, which was not pursued further in the present study (Additional file 3: Figure S1C and D). In summary, FBXO11 specifically fuels tumor formation in non-EMT-like cells by restraining the p53/p21 pathway.

In absence of an obvious EMT-based explanation to tumor aggressiveness, we sought for another mechanism. The most straightforward candidate process for EMT-independent invasion and metastasis is that by collective invasion [10]. Here, we decided to gauge for collective cancer-cell invasion in a basement membrane chamber assay. In an initial experiment we unequivocally demonstrate that deletion of FBXO11 leads to near absence of invasion (Fig. 2j). While this was expected, exactly how the non-EMT-like cells invaded was further investigated by mixing GFP-labeled and non-labeled cells in suspension (Fig. 2k). When the clusters were plated in the invasion assay, cells emerged from single pores as mixed duplets (Fig. 2k), suggesting that indeed non-EMT breast cancer cells spread in a collective manner.

## Conclusion

Based on these observations, it is concluded that FBXO11 is a candidate molecular alternative to the canonical EMT-dependent aggressiveness in highly differentiated luminal tumors. In addition, our results suggest a more prominent role for FBXO11 in subclone-behavior than hitherto anticipated. This emphasizes the relevance of considering clonal heterogeneity in future therapeutic strategies in breast cancer.

## Additional files

Additional file 1: pLKO human shRNA library and its validation. (XLS 1013 kb)

Additional file 2: Screening summary of normalized reads of shRNAs. (XLSX 379 kb)

Additional file 3: **Table S1.** Clinical outcome as a function of FBXO11 expression in breast cancer subtypes and **Figure S1.** FBXO11 facilitates protein degradation in a subclone-dependent manner. (PDF 315 kb)

Additional file 4: Materials and Methods. (DOCX 33 kb)

## Abbreviations

BLI: bioluminescence imaging
CRISPR-Cas9: Clustered regularly interspaced short palindromic repeats-CRISPR associated system 9
d: Day
EMT: Epithelial to mesenchymal transition
ER: Estrogen receptor
FBXO11: F box protein 11
HR: Hazard ratios
hr.: Hour
K: Keratin
NOG: NOD/Shi-*scid*/IL-2Rγ^null^ mouse
RT-qPCR: Reverse transcription-quantitative polymerase chain reaction
SCR: Scrambled shRNA control
SD: Standard deviation
SEM: Standard error of the mean
shRNA: Short hairpin RNA
