# Supplementary material for: Aggressiveness of non-EMT breast cancer cells relies on FBXO11 activity
Source: Mol Cancer. 2018 Dec 10;17:171. doi: 10.1186/s12943-018-0918-6 (PMC6287350; doi:10.1186/s12943-018-0918-6)
Supplement: Supplementary file 3 — Table S1. Clinical outcome as a function of FBXO11 expression in breast cancer subtypes and Figure S1. FBXO11 facilitates protein degradation in a subclone-dependent manner. (PDF 315 kb) [file 12943_2018_918_MOESM3_ESM.pdf]

Table S1. Clinical outcome as a function of FBXO11 expression in breast cancer subtypes

A

Group	No. of PTs	Mean expression of the FBXO11 probes		Relapse Free Survival	
		P value	HR	P value	HR
All	**3951**	**0**	**1.4 (1.25-1.57)**	**1.7e-11**	**1.46 (1.31-1.63)**
LUMINAL A	**1933**	**0.0001**	**1.43 (1.2-1.71)**	**7.1e-5**	**1.43 (1.2-1.71)**
LUMINAL B	**1149**	**0.0003**	**1.44 (1.18-1.75)**	**2e-4**	**1.45 (1.19-1.75)**
BASAL	**618**	**0.0036**	**1.53 (1.15-2-03)**	**3.9e-3**	**1.52 (1.14-2)**
P53 wildtype	273*	0.064	0.65 (0.41-1.03)	0.077	0.67 (0.42-1.05)
P53mutated	188*	0.0057	0.5 (0.3-0.82)	**3.3e-3**	**0.49 (0.3-0.8)**

B

Group	No. of PTs	Mean expression of the FBXO11 probes		Overall Survival	
		P value	HR	P value	HR
All	**1402**	**0.0196**	**1.31 (1.03-1.67)**	**0.0073**	**1.39 (1.09-1.76)**
LUMINAL A	**611**	**0.023**	**1.71 (1.08-2.71)**	**0.0217**	**1.71 (1.08-2.71)**
LUMINAL B	433*	0.76	0.94 (0.65-1.38)	0.87	0.97 (0.67-1.41)
BASAL	241*	0.88	0.96 (0.59-1.58)	0.88	0.96 (0.59-1.58)
P53 wildtype	187*	0.8991	1.04 (0.54-2.02)	0.92	1.03 (0.54-1.96)
P53mutated	111*	0.4522	1.48 (0.53-4.11)	0.89	0.95 (0.42-2.11)

Table S1. Clinical outcome as a function of FBXO11 expression in breast cancer subtypes.

Relapse free survival (RFS) (**A**) and overall survival (OS) (**B**) in different subtypes based on FBXO11 expression. The patients were divided into two groups having high or low expression by a commutated best cutoff. To measure a significance of RFS and OS, p value based on Bonferroni multiple testing correction and hazard ratio (HR) were analyzed. The RFS rate is significantly lower in the FBXO11 high expression group than in the FBXO11 low expression group in luminal A (ESR1+/HER2-/KI67low) and luminal B (ESR1+/HER2-/KI67high or ESR1+/HER2+) as well as basal-like (ESR1-/HER2-) subtypes. This suggests that FBXO11 expression is indeed a poor prognostic factor irrespective of major subtype stratification. FBXO11 expression is a strong predictive indicator of OS of the luminal A subtype. Further, stratification is not feasible due to limited number of patients which are indicated with asterisks in the tables. For the same reason, we cannot conclude on the effects of FBXO11 expression on p53 wild type vs. p53 mutated groups. The statistically significant data are in bold. No. of PTs, number of patients; HR, hazard ratio.

Figure S1. FBXO11 facilitates protein degradation in a subclone-dependent manner

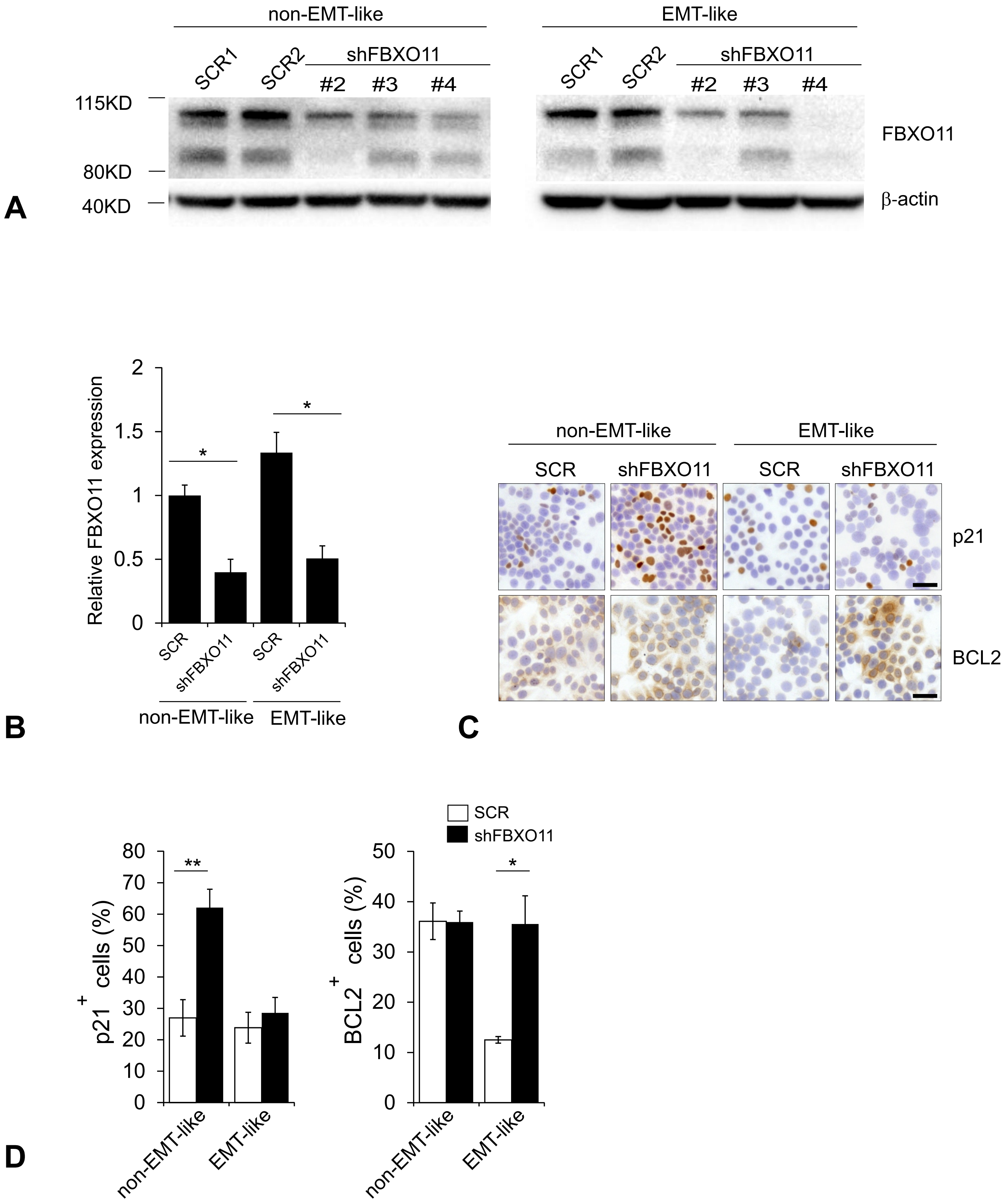

Figure S1. FBXO11 facilitates protein degradation in a subclone-dependent manner. (**A**) Western blots confirm that three different pLKO-shFBXO11 (#2, #3, #4) as compared to two scrambled shRNAs (SCR1, SCR2) inhibit protein expression of FBXO11 in non-EMT-like and EMT-like cells. (**B**) Bar diagram of relative FBXO11 expression measured by RT-qPCR in both cell types showing that shFBXO11 reduces FBXO11 mRNA levels as compared to SCR1 (asterisk indicates $p<0.05$ by t-test). Error bars represent SD of the mean. (**C**) Representative immunocytochemical staining (brown) of p21 (**top row**) and BCL2 (**bottom**) in non-EMT-like (**left**) and EMT-like cells (**right**) transduced with pLKO-SCR or pLKO-shFBXO11 shows that whereas p21 is induced upon FBXO11 inhibition in the non-EMT-like cells but not in the EMT-like cells, BCL2 is induced by shFBXO11 in the EMT-like cells only. Nuclei are counterstained by hematoxylin (blue). Scale bar, 50 μm (**D**) Quantification of immunostaining (brown) of p21 and BCL2. The percentage of immunostained $p21^+$ cells or $BCL2^+$ cells among SCR transduced (white bars) or shFBXO11 transduced (black bars) in a total of approximately 1000 non-EMT-like or EMT-like cells automatically counted with image J in triplicates (asterisks ** and * indicate $p<0.005$ and $p<0.05$ by t-test, respectively). Error bars represent SD.
