# Supplementary material for: Aggressiveness of non-EMT breast cancer cells relies on FBXO11 activity
Source: Mol Cancer. 2018 Dec 10;17:171. doi: 10.1186/s12943-018-0918-6 (PMC6287350; doi:10.1186/s12943-018-0918-6)
Supplement: Supplementary file 4 — Materials and Methods. (DOCX 33 kb) [file 12943_2018_918_MOESM4_ESM.docx]

**Additional file 4: Materials and Methods**

## Cell culture

The non-EMT-like and the EMT-like cells were cloned from MCF7 cells as previously described (1) and cultured in DMEM/F12 medium (Gibco) supplemented with 2 mM Glutamine (Gibco), 50 μg/ml gentamycin (Biological Industries), 6 ng/ml insulin (Sigma), MEM Non-essential Amino Acids Solution (Gibco) and 5% fetal bovine serum (FBS, Sigma).

## Pooled loss-of-function screening

A customized lentiviral short hairpin RNA (shRNA) library, hereafter referred to as epi-library, was established based on the pLKO vector by selecting shRNAs targeting genes that are involved in epigenetic regulation (Sigma). This epi-library consists of 2365 shRNAs targeting 601 different genes (Additional File 1). Twenty million cells were transduced with both the epi-library and the pLKO-GFP virus at a ratio of 1:1. In order to maximize the number of cells with a single shRNA transduced per cell, the amount of virus was titrated to establish total 30% transduction efficiency. The transduction efficiency was confirmed by measuring total GFP expressing cells using fluorescence activated cell sorting (FACS) analysis 2 days after transduction. At day 3, cells were further selected with 1 µl/ml puromycin (Fisher Scientific) and subcultured for additional 17 days. Genomic DNA was extracted at day 3 and at day 20, respectively. A control was also collected with the samples to check for construct representation after transduction. Inserted shRNAs were amplified by polymerase chain reaction (PCR) with primers containing Illumina adaptor sequences and barcode sequences which identify the origins of the samples. The amplified shRNAs were purified by gel extraction (Qiagen) and sequenced by Illumina Hiseq sequencing performed at the Danish National High-throughput DNA sequencing center, University of Copenhagen. Raw reads were trimmed for sequencing adapters using the Cutadapt tool (2). Trimmed reads were aligned to the shRNA libraries using the Burrows-Wheeler alignment (BWA) program (3). A maximum of one mismatch between the sequencing read and an shRNA sequence were allowed. For each shRNA, reads per million (RPM) values were calculated. The list of shRNAs with each RPM is shown in Additional File 2. RPMs were then filtered to select genes whose expression had decreased more than two-fold at day 20 compared to those at day 3.

## Plasmids and viral production

For lentiviral production, HEK293TF cells were transfected with pLKO shRNAs, pCMV-8.9 and pCMV-VSV.G plasmids using the calcium phosphate method. To confirm the screening data, three different pLKO-puro-shFBXO11s were individually co-transfected with packaging plasmids. Similarly, pGIPZ shRNA mir targeting FBXO11 (V2LHS_137108) was co-transfected with packaging plasmids. For *in vivo* imaging, dual function bioluminescent imaging fluorescent reporter pFU-L2G (a gift from Dr. Gambhir, Stanford University) was co-transfected. To create indel mutation of FBXO11 gene, pSpCas9(BB)-A2-Puro (PX-459, a gift from Dr. Zheng, Addgene #62988) was used for cloning and genomic editing was performed by the CRISPR-cas9 system, essentially as described (4). Viral supernatants were collected 48 hours post transfection and filtered through a 0.45 µm filter.

## Animal studies

All experiments were performed with permission from the Animal Experiments (J. nr. 2012-15-2934-00571 and 2017-15-0201-01315). 10^3^ cells (in two independent experiments of n=6 to 10 inoculations in pLKO-shFBXO11 clones, one experiment of n=6 inoculations in pGIPZ-shFBXO11 and in one experiment of n=6 inoculations in each FBXO11 indel clone (F4 or G11) compared to non-EMT-like cells) or 10^4^ cells (in two independent experiments of n=4 injections/group) in a suspension of 50% Matrigel (BD Biosciences) were injected into the 4^th^ mammary fat pad of female NOG mice (Taconic) aged 8 to 12 weeks, and the experiments were run for 6 and 11 weeks, respectively. The drinking water was supplemented with estradiol (0.667 μg/mL, Sigma) and changed once a week. Tumor growth was monitored weekly by palpation. For monitoring tumor growth by bioluminescent imaging in mice, luciferin (10 µl/g mouse wt) was injected intraperitoneally, 10 minutes prior to taking images. The mice were anesthetized in an induction chamber infusing 2.5 to 3% isoflurane (Abbott Laboratories). Bioluminescent imaging was captured by using the IVIS spectrum CT and signals as total flux radiance in photons/sec were quantified using living image 3.0 (Caliper Life Sciences).

## Human breast carcinoma materials

The use of human material has been reviewed by the Regional Scientific Ethical Committees (Region Hovedstaden) and approved with reference to H-2-2010-051 and H-3-2010-095. Human breast carcinoma biopsies (n=30) were obtained from women undergoing mastectomy because of breast cancer, classified as luminal subtypes based on immunostaining for estrogen receptor-α, progesterone receptor and HER2 as previously described (5). Among 30 biopsies, eight biopsies were highly differentiated, polarized grade 1 tubular breast carcinomas. Based on semiquantification of immunostaining for FBXO11 under the light microscopy by two observers, the biopsies were classified into three groups; group 1, less than 10% cancer cells were positive (n=2): group 2, 10 to 50% positive (n=8) and group 3, more than 50% cancer cells were strongly positive (n=18).

## Gene-expression analysis

Total RNA was extracted in Trizol (Invitrogen) according to the manufacturer´s instruction and RNAs were reverse transcribed using the High Capacity RNA-to-cDNA Kit (Applied Biosystems). Quantified real-time PCR was performed using Taqman gene expression assays (Applied Biosystems) on Bio-Rad CFX manager 3.0 and thermocycler (Bio-Rad). Expression of CDH1 (Hs01023894_m1), OCLN (Hs00170162_m1), SNAI2 (Hs00950344_m1), and TWIST (Hs01675818_s1) were normalized with normalized to GAPDH (Hs02758991_g1), TBP1(Hs00427621_m1), TFRC(Hs00951083_m1) and PGK1(Hs00943178_g1). Expression of FBXO11 (Hs00251516_m1) was normalized to GAPDH (Hs02758991_g1), TBP1 (Hs00427621_m1), and TFRC(Hs00951083_m1). Gene expression was calculated by the ΔΔCt method.

## Western blotting

Proteins were extracted by using RIPA lysis buffer (Sigma) including a protease inhibitor cocktail (Sigma P8340) and phosphatase inhibitor cocktails 2 and 3 (Sigma P5726, P0044). Denatured proteins were separated by running on 4–12% Novex™ Bis-Tris pre-cast polyacrylamide gradient gels (Life technologies), transferred onto a PVDF membrane overnight at 0.35A followed by incubation in 5% bovine serum albumin/TBS-Tween blocking buffer. The molecular weight was indicated by using a pre-stained protein ladder (Thermo Scientific, 26616). Primary antibodies included anti-β-actin (1:5000, Sigma A-5441), anti-FBXO11 (1:1000, Novus NB100-59826 and Bethyl laboratories A301-177A), anti-p21 (1:500, Dako M7202) and anti-p53 (1:500, DAKO M7001). Secondary antibodies were conjugated with horseradish peroxidase (HRP): anti-mouse (1:3000, DAKO, P0447) and anti-rabbit (1:3000, DAKO, P0448). Blots were visualized using enhanced chemiluminescence solution (Pierce ECL, Thermo Scientific) in a chemiluminescence imager, Amersham Image 600 (GE Healthcare Life Sciences).

## Immunocytochemistry and histochemistry

Cultured cells, cryosections (6μm) of human breast carcinomas and dissected mouse lung tissue were fixed in 3.7% formaldehyde and in methanol : acetone (1:1), and permeabilized by 0.1% Triton X-100 as described (6). For fluorescent staining, primary antibodies of anti- ZO-1 (1:25, Thermo Fisher, ZO1-1A12), anti-occludin (1:25, Abcam, abB31721), anti-E-cadherin (1:25, HECD1, a gift from Dr. Hirohashi (7)), and anti-K19 (1:50, Biolegend, A53-B/A2) and the corresponding secondary antibodies of Alexa Fluor 488 anti-mouse IgG1 (1:500, Invitrogen), Alexa Fluor 488 anti-rabbit IgG (1:500, Invitrogen), Alexa Fluor 488 anti-mouse IgG1, and Alexa Fluor 568 anti-mouse IgG2a (1:500, Invitrogen) were used. Nuclei were stained with DAPI (Thermo Fisher). Anti-FBXO11 (1:50, Bethyl Laboratories A301-177A), anti-p21 (1:50, Dako M7202), and anti-BCL2 (1:50, Dako, 124) were also used for immunostaining followed by HRP-conjugated universal secondary antibody complex using Ultra Vision One HRP Polymer (Thermo Scientific) and visualized by incubating with 3.3’-Diaminobenzidine solution (DAB, Sigma) activated with 0.02 % hydrogen peroxidase (Merck). Nuclei were counterstained by haematoxylin (Sigma).

## FACS analysis

FACS was used to select pFU-L2G transduced cells or to quantify percentages of GFP^+^ cells as a measurement of proliferation. For the latter purpose, FACS was performed with cells at 20 days after mixed culture of 50% of pGIPZ-GFP-SCR or pGIPZ-GFP-shFBXO11 transduced non-EMT-like cells with 50% of the parental non-EMT-like cells and approximately 10^5^ cells were counted in triplicates using BD FACSDiva software in a flow cytometer (FACS Aria or FACS fusion, BD Biosciences). For FACS analysis, cells were dissociated into single cells using trypsin/EDTA (Life Technologies) or Accutase (Millipore) and passed through a 20 µm filter cup (Falcons). Propidium Iodide (1 µg/ml, Life Technologies) or fixable viability stain 780 (1:1000, BD Biosciences) were used to distinguish living from dead cells.

## Genome editing by the CRISPR-CAS9 system

Single guide RNAs (sgRNAs) were designed by using the online CRISPR design tool (crispr.med.edu) with input sequences of FBXO11 exon 4 (NM_025133.4; NM_001190274, exon 5) which identified as the top target site for sgRNA (CAGGTTCAGGATGCATCATAGGG, referred to as sgFBOX11). sgFBXO11 was cloned into pSpCas9(BB)-A2-Puro as described (4). The sgFBXO11/Cas9 plasmid was transfected into the non-EMT like cells, using lipofectamine 2000 (Thermo Scientific) according to the manufacturer’s instructions. At day three, selection was initiated with 1 μg/mL puromycin and sustained for two days. After two weeks, the cells were single-cell seeded using FACS and clonally expanded. Several clones were tested for deletion of FBXO11 by Western blot analysis. To confirm the target-specific editing, primers were designed to amplify approximately 300 bp on either side of the Cas9- target by PCR (forward primer, CGTTTTCACGTTTTAAA TCCTGTTT; reverse primer, GGAGTGCAGCAGTGCAATG; amplicon 771 bps). The amplicons were sequenced by Sanger sequencing. Untransfected cells or cells transfected with the pSpCas9(BB)-A2-Puro alone were used as a negative controls of the genomic editing.

## Clinical dataset analysis

To validate a prognostic value of FBXO11 expression levels in breast cancer patients, relapse free survival and overall survival at 13 years after surgery were analyzed using an online survival analysis tool (8). The mean expression of three different FBXO11 probes (IDs: 203255_at, 219208_at, 222119_s_at) were used to divide breast cancer cohorts into two groups using the best-cut off setting as determined by the analysis tool. For relapse free survival, 30 datasets of total 3951 patients were used, which include datasets: E-MTAB-365, GSE11121, GSE12093, GSE12276, GSE1456, GSE16391, GSE16446, GSE16716, GSE17705, GSE17907, GSE19615, GSE20271, GSE2034, GSE20685, GSE20711, GSE21653, GSE2603, GSE26971, GSE2990, GSE31519, GSE3494, GSE37946, GSE42568, GSE45255, GSE4611 GSE4922, GSE5327, GSE6532, GSE7390, GSE9195. For overall survival, 10 datasets of total 1402 patients were used and the datasets include GSE1456, GSE16446, GSE16716, GSE20271, GSE20685, GSE20711, GSE3494, GSE37946, GSE42568, GSE45255, GSE7390.

## Invasion assay

Prior to the assay, cells were starved in DMEM/F12 medium with 1% serum for 72 hours. Cells were then detached and separated into single cells using Accutase. A total number of 5x10^4^ cells were seeded in 1% FBS onto Matrigel-coated 24-well FluoroBlok™ inserts with 8 μm pores (Corning) in quadruplicates, and inserts were then transferred onto 10% FBS for 48 hours. At the end point, cells that had migrated were visualized by immunostaining using primary antibodies: anti-K19 (1:250, Abcam, BA16) and anti-GFP (1:250 or 1:500, Invitrogen, A11120) together with secondary antibodies Alexa Fluor 488 anti-mouse IgG2a and Alexa Fluor anti-mouse 568 IgG1(1:500, Invitrogen). Filters were cut from the inserts and mounted on a slide with DAPI containing mounting medium (Invitrogen). Two independent experiments were performed and the total number of invaded GFP^+^ and K19^+^ cells were counted by fluorescence microscopy at 20x magnification. In order to test for collective migration of non-EMT-like cells, cell-clusters were formed prior to invasion assays, by mixing GFP-labeled and non-GFP-labeled cells in 1:1 ratio of total 5x10^4^ cells and cultured in 1% FBS in low-affinity 24-well plates for 24 hours. Clustered cells were then transferred to the Matrigel-coated inserts and incubated for 72 hours. Three independent experiments were performed in duplicate.

## Statistical analysis

All the statistical analyses were tested using a statistical computing and graphic program R (version 3.3.2). Applied statistical tests are indicated in the figure legends.

1. Kim J, Villadsen R, Sørlie T, Fogh L, Grønlund SZ, Fridriksdottir AJ, et al. Tumor initiating but differentiated luminal-like breast cancer cells are highly invasive in the absence of basal-like activity. Proc Natl Acad Sci USA. 2012;109(16).

2. Marcel M. Cutadapt removes adapter sewuences from high-throughput sequencing reads. EMBnetjournal. 2011;17(1):10-2.

3. Li H, Durbin R. Fast and accurate short read alignment with Burrows-Wheeler transform. Bioinformatics. 2009;25:1754-60.

4. Ran FA, Hsu PD, Wright J, Agarwala V, Scott DA, Zhang F. Genome engineering using CRISPR-Cas9 system. Nat Protoc. 2013;8:2281-308.

5. Balk-Møller E, Kim J, Hopkinson B, Timmermans-Wielenga V, Petersen OW, Villadsen R. A marker of endocrine receptor-positive cells, CEACAM6, is shared by two major classes of breast cancer: luminal and HER2-enriched. Am J Pathol. 2014;184(4):1198-208.

6. Hopkinson BM, Klitgaard MC, Petersen OW, Villadsen R, Rønnov-Jessen L, Kim J. Establishment of a normal-derived estrogen receptor-positive cell line comparable to the prevailing human breast cancer subtype. Oncotarget. 2017;8:1-14.

7. Shimoyama Y, Hirohashi S. Expression of E- and P-cadherin in gastric carcinomas. Cancer Res. 1991;51:2185-92.

8. Györffy B, Lanczky A, Eklund AC, Denkert C, Budczies J, Li Q, et al. An online survival analysis tool to rapidly assess the effect of 22,277 genes on breast cancer prognosis using microarray data of 1,809 patients. Breast Cancer Res Treat. 2010;123:725-31.
